# VEGF Upregulation in Viral Infections and Its Possible Therapeutic Implications

**DOI:** 10.3390/ijms19061642

**Published:** 2018-06-01

**Authors:** Khaled R. Alkharsah

**Affiliations:** Department of Microbiology, College of Medicine, Imam Abdulrahman Bin Faisal University (IAU), P.O. Box 1982, Dammam 31441, Saudi Arabia; kalkharsah@iau.edu.sa; Tel.: +966-13-3331053

**Keywords:** angiogenesis, vascular endothelial growth factor (VEGF), vascular endothelial growth factor receptor (VEGFR), Epstein-Barr virus (EBV), Kaposi’s sarcoma herpesvirus (KSHV), Hepatitis C Viruses (HCV), Hepatitis B Viruses (HBV), Herpes Simplex Virus (HSV), Hantavirus, Dengue fever virus, therapeutics

## Abstract

Several viruses are recognized as the direct or indirect causative agents of human tumors and other severe human diseases. Vascular endothelial growth factor (VEGF) is identified as a principal proangiogenic factor that enhances the production of new blood vessels from existing vascular network. Therefore, oncogenic viruses such as Kaposi’s sarcoma herpesvirus (KSHV) and Epstein-Barr virus (EBV) and non-oncogenic viruses such as herpes simplex virus (HSV-1) and dengue virus, which lack their own angiogenic factors, rely on the recruitment of cellular genes for angiogenesis in tumor progression or disease pathogenesis. This review summarizes how human viruses exploit the cellular signaling machinery to upregulate the expression of VEGF and benefit from its physiological functions for their own pathogenesis. Understanding the interplay between viruses and VEGF upregulation will pave the way to design targeted and effective therapeutic approaches for viral oncogenesis and severe diseases.

## 1. Introduction

Angiogenesis is an important physiologic process which involves formation of new blood vessels from already existing vasculature. It plays a vital role during development and wound healing but also during disease pathology such as tumor growth and progression. Several stimulators are involved in the angiogenesis process including vascular endothelial growth factor (VEGF), which plays a crucial role in activating endothelial cells through binding to receptors on the cell surface called vascular endothelial growth factor receptor (VEGFR) [1]. There are four VEGF isoforms in mammals (VEGF-A,B,C,D) and the placental growth factor (PlGF) encoded by different but related genes [2]. Most of these isoforms express alternative splice variants or proteolytic cleaved proteins to produce a range of functional VEGF isoforms and splice variants. Furthermore, VEGF isoforms signal through different receptors (VEGFR1,R2 and R3) and coreceptors including the neuropilin receptors (Nrp1 and 2), heparan sulfate and integrins [3]. The three VEGFRs are tyrosine kinases and mediate signal transduction upon ligand binding and dimerization [4]. VEGFR1 binds VEGF-A, VEGF-B and PlGF. It is also present in a soluble form and therefore expected to play a negative regulatory role in VEGF signaling [5]. VEGFR2 binds VEGF-A, VEGF-C and VEGF-D and is implicated in most vascular and endothelial biological processes through phosphorylation activities at several tyrosine residues in its cytoplasmic tail [6]. VEGFR3 is the receptor for VEGF-D and VEGF-C and expected to play a role in lymphatic endothelium development [6]. Nonetheless, VEGFR3 was found to additionally play a role through its kinase activity in sprouting of endothelial cells during blood vessels formation [7,8].

VEGF-A is the prototype of all VEGFs and by far the most extensively studied isoform and therefore in some literature is referred to as VEGF. Seven proangiogenic splice variants exist from VEGF-A based on the presence of alternative splice site selection in exons 6 and 7 generating a variety of isoforms partially or completely lacking exons 6, 7 or 8 [9,10]. This family of splice variants is called VEGF-Axxx, where the triple-x indicates the number of amino acids in the protein. Another family of VEGF-A splice variants is produced based on an extra splicing site in exon 8 in addition to the splicing sites in exon 6 and 7. The involvement of the distal exon 8 splicing site in VEGF-A production generates VEGF isoforms similar to their VEGF-Axxx counterparts but with six different amino acids at its carboxy-terminus. This family of splice variants is called VEGF-Axxxb and is believed to have antiangiogenic properties [9,10].

**Figure 1:**
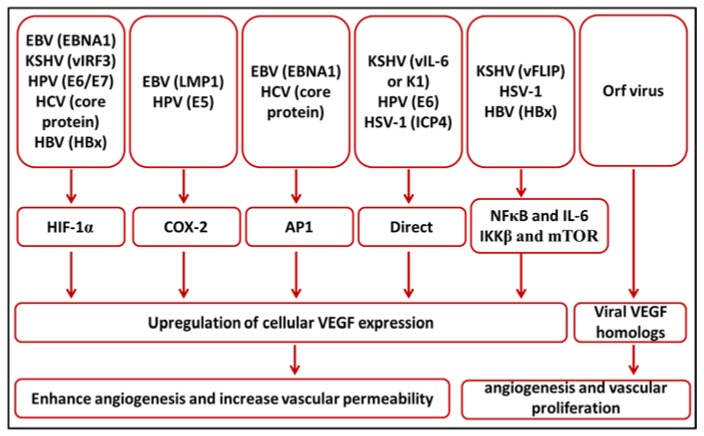
A schematic diagram summarizes how different viruses mediate vascular endothelial growth factor (VEGF) upregulation.

Human viruses cause a variety of diseases where neither specific successful treatment nor safe and effective vaccine are available. VEGFs and their receptors have been implicated in the pathophysiology of many of these diseases. This review discusses the involvement of VEGFs and their receptors in viral diseases and the possible therapeutic applications. Table 1 and Figure 1 summarize common human viruses and their diverse mechanism of upregulation of VEGF expression.

## 2. Upregulation of VEGF Expression in Viral Oncogenesis

### 2.1. Epstein-Barr Virus (EBV)

EBV is a human herpesvirus that asymptomatically infects the majority of the human population. EBV infection has been implicated in many diseases such as infectious mononucleosis after primary infection and neoplasia including lymphoproliferative disorders and lymphomas such as Burkitt’s lymphoma, nasal NK/T-cell lymphoma and a subset of Hodgkin’s lymphoma [11]. EBV was also found to be associated with solid tumors such as nasopharyngeal carcinoma (NPC) and small fraction of gastric carcinoma [12,13]. During infection, EBV establishes a state of latency where only few viral proteins are expressed driven by the pressure from the immune system.

VEGF expression and microvascular density were shown to directly correlate with NPC and its metastatic progression, indicating the importance of VEGF for NPC growth and progression [14]. Several other studies found a correlation between the severity of NPC or its metastatic progression and the levels of VEGF in circulation, saliva or in the tumor itself [15,16,17,18]. The EBV latent membrane protein 1 (LMP1) is one of the proteins expressed during the latency state II of EBV and is frequently detected in NPC along with EBNA1, LMP2A and B, and a transcript from *Bam*HI A restriction fragment (BART) [19]. The overexpression of the oncoprotein LMP1 in transgenic mice led to upregulation of VEGF expression early in life accompanied with hyperplasia and increased vascularization which progressed spontaneously later to carcinoma [20]. LMP1 is a transmembrane protein and seems to exert its effect on VEGF expression through other mediators such as the JNKs/c-Jun signaling [21]. Furthermore, LMP1 expression was found to be concomitant with COX-2 expression in NPC. Overexpression of LMP1 in nasopharyngeal epithelial cell lines increased the expression of COX-2, which in turn enhanced VEGF expression [22]. EBV-LMP1 expression was also found to be significantly associated with VEGF expression in diffuse large B cell lymphoma (DLBCL) and their expression was associated with reduced survival rate [23].

EBNA1, an EBV nuclear protein, plays an important role in virus persistence and was demonstrated to be expressed in EBV-associated tumors. EBNA1 seems to play an additional role in EBV-associated malignancies by indirect enhancement of angiogenesis through the activation of AP-1 transcription factor in NPC [24]. AP-1 expression is enhanced by EBNA1 binding and consequently leads to increased expression of its downstream targets such as VEGF and IL-8 [24].

EBV is associated with a small fraction of gastric carcinoma cases and was associated with increased alteration in the PI3K pathway [25]. Several viral sequences were detected in the tumor cells including EBNA1. VEGF expression is upregulated in EBV-associated gastric cancer and influenced by the overexpression of hypoxia-inducible factor-1 alpha (HIF-1α) [26]. LMP1 is known to directly induce the expression of the HIF-1α [27]; however, LMP1 is not expressed in EBV-associated gastric carcinoma. Whether EBV achieves the upregulation of VEGF through EBNA1 in gastric carcinoma needs to be elucidated.

It is obvious that EBV upregulates the expression of VEGF via its own oncoproteins, LMP1 and EBNA1, and exploits the VEGF proangiogenic characteristics to promote the growth and progression of its tumors. EBV is a ubiquitous virus and infects a large proportion of the human population. The oncoproteins, LMP1 and EBNA1, are expressed in most EBV-infected cells yet and luckily only a small number of EBV infections progress to tumor. What are the major factors involved in EBV-associated tumorigenesis are not clearly identified but many host genetic factors and environmental risk factors are expected to play an additional role.

### 2.2. Kaposi’s Sarcoma-Associated Herpesvirus (KSHV)

Kaposi’s sarcoma (KS) is a malignancy characterized by neoangiogenesis and infiltration of inflammatory cells. It is also characterized by the presence of spindle cells which were all found to be infected by the Kaposi’s sarcoma-associated herpesvirus (KSHV) [28,29]. KSHV was detected in all forms of KS and is associated with two other neoplasms, the primary effusion lymphoma and multicentric Castleman disease. KS lesions express large amounts of VEGF which is important for the tumor growth, while blocking of the VEGF receptors abolishes tumor growth [28,30,31]. Moreover, VEGFR2 was shown to be upregulated in the tumor endothelial cells and the stromal vessels in tumor and tissues surrounding it [32,33]. Animal experiments provided an insight into the role of KSHV genes in VEGF upregulation. KSHV-infected endothelial cells when injected into nude mouse promoted tumor formation with expression of elevated levels of VEGF [34]. In another mouse model, bone marrow-derived endothelial cells were infected with KSHV-genetically engineered in a bacterial artificial chromosome (BAC) and injected into severe combined immunodeficiency (SCID) mice [35]. The mice developed tumor reminiscent of KS and expressed increased levels of VEGF [35].

Similar to EBV, KSHV is present in KS spindle cells in a latent form and only few genes are expressed. The viral FLICE inhibitory protein (vFLIP) encoded by KSHV *ORFK13* is one of these genes, which is responsible for the spindling morphology of KS endothelial cells [36,37]. vFLIP activates the canonical and noncanonical NF-κB pathways and hence it is responsible for the inflammatory profile observed in KS lesion [38,39]. The expression of inflammatory mediators and growth factors induced through vFLIP activation of NF-κB attracts the migration and recruitment of inflammatory cells such as monocytes and macrophages, which constitute a generous source of VEGF production [40].

Other KSHV proteins were implicated in VEGF expression directly such as K1 and viral interlukin-6 (vIL-6) [41,42]. The viral G-protein coupled receptor (vGPCR) was reported to produce KS-like lesions with high VEGF levels in transgenic mice [43]. The viral interferon regulatory factor 3 (vIRF3) was found to enhance the stability of HIF-1α to induce VEGF expression [44]. It is worth noting that all of these proteins are considered proteins of the lytic KSHV replication cycle and not expressed in KS lesions where KSHV is mainly latent. However, it was demonstrated that a minor number of KSHV-infected cells undergoes spontaneous virus lytic replication [45]. This small fraction of cells provides continuous virus supply to infect new cells and provides a wide spectrum of viral genes that are involved in inflammatory profile of the KS lesion.

### 2.3. Human Papillomavirus (HPV)

HPV infects the mucosal and cutaneous tissues and is the common cause of warts on skin and genitalia. In 1970, HPV was identified in cervical cancer and a decade later in a subset of oropharyngeal carcinomas (OPC) [46]. Since then, HPV was implicated in the pathogenesis of many human cancers at variable percentages including cervical cancer, anal cancers, penile cancers, vaginal cancers, vulvar cancers, head and neck carcinoma, and skin cancer [47]. About 179 genotypes of HPV are known so far based on variation in their genome sequence [48]. HPV genotypes can be classified into low risk and high risk based on their malignancy transforming capability. Those genotypes implicated in HPV-associated malignancies are considered high risk and these are mainly HPV16 and HPV18; however, other genotypes such as HPV31, HPV33, HPV35, HPV39, HPV45, HPV51, HPV52 and HPV56 are being frequently reported in cancer [48,49,50]. HPV encodes 10 proteins, three of which (E5, E6 and E7) are detected in human tumors and considered to be responsible for oncogenesis [51]. E5 protein on its own has a weak transforming activity in cell culture but it can collaboratively potentiate the transforming activity of both E6 and E7 [52]. The transforming magnitude of HPV lies in E6 and E7 proteins where both are able to enhance cell proliferation, destabilize the genome and more importantly abrogate apoptosis [51]. E6 utilizes the cellular ubiquitin ligase E6AP to target p53 for degradation [53]. E7 activity leads to activation of the elongation factor 2 (E2F) and increased expression of the cellular p16 and ultimately enhance cell proliferation [54]. Further details on the role of E6 and E7 in HPV oncogenesis can be found in [51].

E6 and E7, especially of HPV16 and HPV18 genotypes, also play a role in VEGF upregulation and therefore by establishing the angiogenic structure of HPV-associated cancers. HPV16 E6 and E7 oncoproteins were demonstrated to upregulate the expression of HIF-1α and VEGF in non-small cell lung carcinoma and cervical carcinoma cells [55,56,57]. Using inhibitors that target the ERK1/2 or PI3K pathways leads to complete inhibition of HIF-1α and VEGF expression, suggesting the involvement of these signaling pathways in E6- and E7-mediated angiogenesis [56]. E6 seems to have a direct effect on the VEGF promoter. It binds to a responsive region consisting of four SP-1 sites in the VEGF proximal promoter region [58]. While E7 exerts its effect on upregulation of VEGF expression through the telomerase reverse-transcriptase (hTERT) and telomerase activity [59], E5 also induces the expression of VEGF. E5 activates the EGFR which in turn leads to the phosphorylation of downstream molecules PI3K and Akt. These enhance the transcription of COX-2, through increasing its promoter activity, leading to increase in VEGF expression [60]. This tight controlled upregulation of VEGF by the three oncoproteins of HPV indicates the importance of VEGF for HPV-associated malignancies and makes it an attractive target for therapeutic approaches.

### 2.4. Hepatitis C Viruses (HCV)

HCV infects a large number of the human population and in the majority of cases the infection is chronic, leading to frequent inflammation which ends up with cirrhosis in about 20% of cases and with hepatocellular carcinoma (HCC) in up to 5% of cases [61]. HCV infection constitutes a risk for development of HCC whose incidence is increasing in many countries [62]. In addition to chronic inflammation, HCV may also directly potentiate the risk of developing HCC by regulating several host pathways including those involved in angiogenesis. HCV proteins (core, NS3, NS5A, NS5B and E) through interference with cellular factors (Rb, Cyclin D, Cyclin E, RAF/MAP/ERK) stimulate cell cycle progression, enhance cellular proliferation, and inhibit apoptosis, which are key players in tumorigenesis [61].

Similar to other tumors, angiogenesis in HCC is critical for tumor growth and progression. HCV was found to be associated with higher microvessel density in HCC [63]. Several studies have shown the upregulation of the proangiogenic factor VEGF in HCV-related HCC tissues, patient’s serum and in cell culture experiments [64,65,66]. Polymorphisms in the VEGF gene were found to increase the VEGF expression levels and to be associated with higher risk of HCC [66]. It is also suggested that VEGF serum levels could be utilized as a prognostic factor of HCC [64,65]. The HCV core protein seems to be the major moderator of HCV-dependent VEGF upregulation in different mechanisms. The core protein stabilizes HIF-1α, which automatically upregulates the expression of VEGF [67,68,69]. Inhibition of the Jak/Stat pathway was found to abrogate the core protein-mediated activation of the androgen receptor and, thereby, the downregulation of VEGF expression, which suggests a role for Jak/Stat signaling pathway in HCV-mediated VEGF expression [70]. Recently it was shown that the core protein also activates the AP-1 transcription factor, which potentiates VEGF expression through direct binding to its promoter [71].

### 2.5. Hepatitis B Viruses (HBV)

HBV is responsible for about half of HCC cases worldwide [72,73]. Contrary to HCV, HBV is a DNA virus; therefore, it can integrate itself in the cellular genome resulting in deletions and general instability [74]. Additionally, HBV establishes chronic infection in 15–40% of cases leading to continuous cycles of necrotic inflammation leading to cirrhosis and eventually to HCC [72]. Similar to other cancers, VEGF seems be the important angiogenic factor of the HBV-related hepatocarcinogenesis. The gene expression profile in HBV-related HCC mouse model showed clear induction of VEGF- and EGF-mediated pathways [75]. VEGF expression was found to be upregulated along with COX-2 in tissue sections from human HCC with HBV infection and this expression was found to be positively correlated with microvessel density (MVD) [76,77].

The hepatitis B viral protein x (HBx) is the key inducer of VEGF expression in HBV-related HCC. VEGF transcription was found to be induced in HBx stably transfected cells [78]. Similar to the HCV core protein, HBx stabilizes HIF-1α and enhances the increase in VEGF expression [79,80,81]. Overexpression of HBx in hepatoma cells leads to induction of mTOR and IKKβ, which in turn enhance cell proliferation and increase the expression of VEGF [82]. Pre-S protein was also reported to potentiate the expression of VEGF and, by that, augment the angiogenic environment produced by the virus [83].

The availability and accessibility of safe and effective recombinant HBV vaccine will markedly decrease the number of new HBV cases and thereby the HBV-related HCC. According to the World Health Organization (WHO), HBV vaccine was incorporated in the infant immunization programs of 95% of countries and about 50% of countries had adopted the recommended dose at birth [84].

## 3. Upregulation of VEGF Expression in Non-Oncogenic Viral Infections

### 3.1. Herpes Simplex Virus-1 (HSV-1)

HSV type 1 infects more than half of the human population and the rate of infection may reach up to 90% in some geographical areas [85]. It causes Herpes labialis (cold sores) after primary infection and then establishes a lifelong infection residing latently in the trigeminal ganglia. HSV-1 may also cause ocular infection called herpetic stromal keratitis (HSK), where the cornea loses transparency due to neovascularization, which results in impaired vision and blindness [86]. VEGF and its VEGFR2 receptor are involved in the pathogenesis of HSK through the induction of lymphatic neoangiogenesis in the cornea and the underlying stromal tissue [87]. HSV-1 infection of the cornea triggers VEGF expression in the cornea and the stromal cells despite the absence of HSV-stromal cells infection [88]. It is believed that VEGF expression in the stroma is mediated by a paracrine effect of the IL-6 cytokine, which is also induced by HSV-1 infection of the cornea [89]. The infection cellular protein-4 (ICP4) seems to be the major player in HSV-mediated VEGF expression. ICP4 binds directly to the proximal promoter of the VEGF gene and drives the transcription of VEGF mRNA in collaboration with other early viral proteins [90]. HSV-1 infection of the cornea does not only upregulate VEGF expression, but also disrupts the balance with its soluble neutralizing receptor (soluble vascular growth factor receptor-1, sVEGFR1) by facilitating the sVEGFR1 degradation via the metalloproteases enzymes which are produced by the infiltrating inflammatory cells as a result of infection [91]. HSV-1 additionally exploits host factors such as the host microRNA-132 and the cytokine IL-17A to disturb the balance between VEGF and its neutralizing receptor sVEGFR1 and render the immune-privileged corneal tissue accessible to inflammatory cells and mediators and induces neovascularization [92,93]. It was recently observed that the fibroblast growth factor-2 (FGF-2), whose expression is also upregulated after HSV-1 infection, sustains the VEGF-mediated neovascularization of the cornea even after resolving of HSV-1 infection [94].

### 3.2. Dengue Virus (DENV)

Dengue virus is an arthropod-borne virus transmitted to human through the Aedes mosquito vector. There have been four serotypes identified (DENV1 to DENV4). DENV causes a mild feverish disease called Dengue fever (DF). However, the disease may develop into severe complications called dengue hemorrhagic fever (DHF), which is characterized by increased capillary permeability and plasma leakage [95]. Continuous plasma leakage may lead to decreased intravascular volume and hypotensive shock (dengue shock syndrome, DSS). VEGF was previously called vascular permeability factor (VPF) as it caused increase permeability of capillaries [96]. Many studies have shown the presence of elevated serum levels of VEGF in patients with DHF but not DF [97,98,99,100,101]. Other studies have demonstrated that DENV infection of pulmonary endothelial cell lines upregulates the expression of VEGF along with many Th1 and Th2 cytokines [102,103]. For this reason, it is believed that immune preparedness is a major determinant of disease severity [95].

### 3.3. Hantaviruses

The *Hantavirus* genus belongs to the family *Bunyaviridae* and comprises a group of zoonotic viruses (such as Andes virus, Hantaan virus, Seoul virus, and others), which transmit from the primary reservoir to human through inhalation of aerosols from rodents’ feces, urine or saliva [104]. Rodents have been identified as the primary reservoir for several Hantaviruses from different geographical regions [105]. Hantaviruses cause serious diseases called Hantavirus Pulmonary Syndrome (HPS) and Hemorrhagic Fever with Renal Syndrome (HFRS) with fatality rate of up to 40% [104,105]. Disease manifestations are overlapping and characterized by increased permeability and vasodilatation which leads to extravasation of inflammatory mediators and blood in the affected organs. High levels of VEGF were implicated in the pathogenesis of many Hantaviruses [106,107].

Andes virus for example infects pulmonary endothelial cells in vitro and induces VEGF expression which leads to abnormal increased permeability [108]. VEGF expression in Andes-infected endothelial cells was found to be preceded by production of virus progeny [109]. Andes virus and Hantaan virus disturb the assembly of adherence junction of vascular endothelial cells by internalizing cadherins of endothelial cell junction and dysregulating β3-integrin and therefore allow extravasation of blood and inflammatory mediators [107,110]. Several other studies showed the upregulation of the VEGF by one or another virus from the *Hantavirus* genus [111,112].

## 4. Viral VEGF Homolog Proteins

Some viruses encode their own proangiogenic homolog such as the VEGF-E encoded by orf virus (ORFV). ORFV belongs to the genus *Parapox* of the family *Poxviridae*. It infects keratinocytes and causes pustular skin disease in sheep and goats which may transmit to human through direct contact [113]. Tissue sections from the ORFV skin lesions show high vascularization and infiltration of inflammatory components [114]. Considering the vascularized and edematous nature of the ORFV lesion, it was tempting to imagine the involvement of VEGF in ORFV pathogenesis. In 1994, it was discovered that ORFV encodes a homolog of the human VEGF later called VEGF-E and was found to be responsible for the virus-associated angiogenesis [113,115]. ORFV lacking functional *VEGF-E* gene causes lesions without dermal swelling and vascular proliferation [116,117]. VEGF-E shares about 25% homology with VEGF-A and binds with strong affinity to VEGFR2 [118,119]. VEGF-E lacks hairpin-binding domain and, therefore, cannot bind and engage the coreceptor heparan sulfate, whereas the VEGF-E variant encoded by the ORFV_NZ2_ was found to bind to the coreceptor neuropilin-1 (NRP-1) through the RPPR peptide in its carboxyterminus and to induce the assembly of VEGFR2-NRP-1 complex [120,121,122]. VEGF-E can induce a strong angiogenic response comparable to VEGF-A, however, without the hemorrhagic effect and without disturbing the endothelial junctions which are considered adverse side effects of VEGF-A angiogenic properties [118]. This favorable angiogenic property of VEGF-E makes it a good candidate for proangiogenic therapy in clinical practice (discussed below).

Other viruses from the *Parapox* genus were found also to express VEGF homolog such as the Bovine papular stomatitis virus (BPSV) and Pseudocowpox (PCPV), which may also infect human [123,124]. A VEGF homolog was also detected in the Parapoxviruses of red deer in New Zealand (PVNZ); however, this virus was not reported to infect human [125].

## 5. Therapeutic Applications of Targeting VEGF in Viral Diseases

Apparently, targeting the molecular modulators of angiogenesis, in particular VEGF, is an attractive area of research and tempting approach for drug design to treat viral oncogenesis and other viral diseases when angiogenesis is involved. Lack of specific treatment and effective vaccination for most virus diseases adds more significance to this approach.

A bulk of evidence shows that the inhibition of VEGF function in viral oncogenesis and viral diseases leads to very promising outcome [126,127]. The encouraging results from inhibition of VEGF-mediated angiogenesis in tumors led to testing anti-VEGF antibody in phase II multicenter clinical trial [128]. It was found that addition of anti-VEGF monoclonal antibody (Bevacizumab) to the traditional chemoradiation treatment of NPC apparently delayed the progression of distant metastasis [128]. The use of rapamycin and other mTOR inhibitors clearly reduced the secretion of VEGF and led to inhibition of KS growth and formation of neovasculature [129]. Recently two clinical phase II trials were performed to test the efficiency of targeting VEGF in cervical neoplasia and NPC. The first study investigated the use of celecoxib on cervical intraepithelial neoplasia 3 (CIN 3) and found that histologic regression rate was only observed in patients with high levels of serum VEGF [130]. On the other hand, testing cetuximab and pemetrexed in combination with radiation therapy showed a promising efficacy with the expected toxicity from these two drug combinations regardless of HPV positivity status [131]. Furthermore, the reduction of VEGF levels by melatonin enhanced the therapeutic potency of the HPV DNA vaccine through potentiating the immune response and production of HPV-E7-specific CD8+ cells [132].

The high vascular nature of HCC made the anti-angiogenic therapies an attractive approach for treatment. Sorafenib, for example, was one of the first anti-angiogenic drugs, which showed improved survival of patients with advanced HCC. Sorafenib is an inhibitor of several tyrosine kinases including the VEGF receptors and was shown to induce apoptosis in cell lines from HCC and inhibit angiogenesis in HCC mouse model [133]. The promising therapeutic effects of sorafenib lead to development of other anti-angiogenic agents that were tested in phase II and III clinical trials alone or in combination and showed promising success in reduction of tumor growth and improving survival in the presence or absence of HCV [134]. Interestingly, sorafenib has an additional inhibitory effect on multiple steps of HCV replication [135,136]. In HBV-related HCC treated with sorafenib, high HBV load was associated with poor prognosis unless an anti-viral therapy was added to the therapy [137].

Targeting VEGF and its receptors in herpetic stromal keratitis by local application of siRNA or with a delivery vehicle in mouse models markedly reduced neovascularization and proved a useful therapy approach for angiogenesis-related ocular diseases [138]. The use of anti-VEGF such as bevacizumab or ranibizumab, along with other therapy techniques including surgery and the use of immunosuppressive drugs, enhanced the restoration of the cornea in herpetic keratitis [139].

The late appearance of the symptoms in Hantavirus Pulmonary Syndrome (HPS) makes antiviral treatment such as interferon and ribavirin ineffective against the disease [140,141]. Therefore, treatment strategies stabilizing endothelial cell permeability and tissue vasculature seem the potential approach to reduce disease severity and mortality [140]. In line with this suggestion, it was observed that the angiopoietin 1 (Ang-1) and sphingosine 1-phosphate (S1P) inhibit endothelial cell permeability induced by Hantavirus [107]. Using the VGEFR2 inhibitor (pazopanib) in addition to the src kinase inhibitor (dasatinib) dramatically inhibited endothelial cell permeability induced by ANDV [142]. Similarly, the use of vandetanib as an inhibitor of VEGFR2 phosphorylation reduced VE-cadherin degradation and modestly increased the survival in HPS animal model [143].

It is worth noting that despite the initial expectation of successful anti-angiogenic therapies, limitation appeared quickly represented by initial response then quick development of resistance. Therefore, and as shown by other studies, anti-angiogenic therapeutic approaches should be used in combination with other drugs that target additional pathway in the disease pathology. Furthermore, studies on therapeutic applications of VEGF and other angiogenic factors in viral malignancies and viral severe diseases should be directed against the viral protein which enhances the production of VEGF rather than cellular VEGF itself and other modulator of angiogenesis. Such viral targets are limited in number, provide good selective targeting approach, and avoid the disturbance of the normal physiologic functions of cellular protein. For example, the use of an LMP1 antibody in combination with the classical chemotherapy showed a marked reduction in VEGF and apoptosis and inhibited NPC xenograft growth in nude mice [144].

VEGF therapeutic applications may extend beyond the inhibition of angiogenesis to promotion of angiogenic effects in clinical practice. The favorable prongiogenic properties of the VEGF-E without enhancement of inflammation and vascular permeability which are common characters of other VEGFs suggest a possible application for VEGF-E in pro-angiogenic therapies [145]. A chimeric protein consisting of VEGF-E and the human PlGF-enhanced vascularization in ischemic tissue [146]. Animal experiments in equine found that the use of Orf virus VEGF-E and IL-10 promotes wound healing and reduces inflammation but has no effect on the speed of wound closure process [147].

## 6. Summary and Conclusions

VEGF seems to be an important player in the pathogenesis of many viral diseases. Therefore, many viruses seek the upregulation of VEGF by several means and some viruses bring their VEGF homolog with them to the infected host (Figure 1). HIF-1α, COX-2 and AP1 appear to be the most common target pathways for virus-mediated upregulation of VEGF. However, some other viruses activate certain inflammatory mediators which end up by the upregulation of VEGF expression. Other viruses directly activate the VEGF promotor to enhance its expression by their own effector proteins (Figure 1). Therefore, major research efforts are required for a very good understanding of the role of viral gene products in upregulation of VEGF expression and are essential for designing novel therapeutic protocols and discovering new chemicals that selectively target viral genes and spare cellular physiologic functions.

## Figures and Tables

**Table 1:** Summary of viruses exploiting vascular endothelial growth factor (VEGF) upregulation in human diseases.

Virus	Disease	Mechanism of VEGF Upregulation	References
EBV	Nasopharyngeal carcinoma	LMP1 upregulates the expression of VEGF through the phosphorylation of JNKs/c-Jun signaling pathways	[20,21]
		LMP1 upregulates COX-2 expression which leads to the upregulation of VEGF expression	[22]
		EBNA1 activates the AP-1 transcription factor which enhances the transcription of VEGF	[24]
	Gastric carcinoma	Overexpression of HIF-1α leads to the upregulation of VEGF expression	[26]
KSHV	Kaposi’s sarcoma	vFLIP activates the transcription factor NF-κB which induce inflammatory response leads to upregulation of VEGF	[38,39,40]
		Viral K1 and vIL-6 directly implicated in expression of VEGF	[41,42]
		vGPCR enhances the upregulation of VEGF	[43]
		vIRF3 stabilizes HIF-1α to enhance VEGF expression	[44]
HPV	Cervical cancer, head and neck carcinoma	E6 binds to a responsive region consisting of four SP-1 sites in the VEGF promoter region	[58]
		E7 upregulates VEGF expression through the telomerase reverse-transcriptase (hTERT) and telomerase activity	[59]
		E5 activates the EGFR which in turn leads to the phosphorylation of PI3K and Akt, enhancement of the transcription of COX-2 leading to increase in VEGF expression	[56,60]
HCV	Hepatocellular carcinoma	Core protein stabilizes HIF-1α which automatically upregulates the expression of VEGF	[67,68,69]
		Core protein activates the AP-1 transcription factor which potentiates VEGF expression through direct binding to its promoter	[71]
HBV	Hepatocellular carcinoma	HBx protein stabilizes the HIF-1α and enhances VEGF expression	[79,80,81]
		HBx induces mTOR and IKKβ which in turn induces VEGF expression	[82]
		Pre-S protein potentiates the expression of VEGF	[83]
HSV-1	Herpetic stromal keratitis	ICP4 binds directly to the proximal promoter of VEGF gene and drives its transcription	[90]
		HSV-1 disturbs the balance between VEGF and its neutralizing receptor sVEGFR1	[91]
DENV	Dengue hemorrhagic fever, dengue shock syndrome	Upregulation of Th1 and Th2 cytokines and VEGF	[102,103]
Hantaviruses	Hantavirus Pulmonary Syndrome, Hemorrhagic Fever with Renal Syndrome	Mechanism jet to be elucidated	
Orf virus	Pustular skin disease	Encodes VEGF homolog called VEGF-E	[113,115]

## Abbreviations

AP-1	Activator protein 1
COX-2	Cyclooxygenase-2
DENV	Dengue virus
E5, E6, E7	HPV early proteins 5, 6 and 7
EBNA1	EBV nuclear antigen 1
EBV	Epstein-Barr virus
EGFR	Epidermal growth factor receptor
HBV	Hepatitis B virus
HBx	HBV-x protein
HCV	Hepatitis C virus
HIF-1α	Hypoxia inducible factor-1 alpha
HPV	Human papilloma virus
HSV-1	Herpes simples virus-1
hTERT	Human Telomerase reverse transcriptase
ICP4	Infected cell protein 4
JNK	c-Jun N-terminal kinases
KSHV	Kaposi’s sarcoma-associated herpesvirus
LMP1	latent membrane protein 1
NF-κB	Nuclear factor kappa B
NPC	Nasopharyngeal carcinoma
Npr	Neuropilin receptor
PI3K	Phosphatidylinositol 3-kinase
PlGF	Placental growth factor
SP-1	Specificity protein 1
VEGF	Vascular endothelial growth factor
VEGFR	Vascular endothelial growth factor receptor
vFLIP	Viral FLICE inhibitory protein
vGPCR	Viral G-protein coupled receptor
vIRF3	Viral interferon regulatory factor 3
